# Immunological responses following administration of a genotype 1a/1b/2/3a quadrivalent HCV VLP vaccine

**DOI:** 10.1038/s41598-018-24762-9

**Published:** 2018-04-24

**Authors:** D. Christiansen, L. Earnest-Silveira, B. Chua, P. Meuleman, I. Boo, B. Grubor-Bauk, D. C. Jackson, Z. Y. Keck, S. K. H. Foung, H. E. Drummer, E. J. Gowans, J. Torresi

**Affiliations:** 1 Department of Microbiology and Immunology, The Peter Doherty Institute for Infection and Immunity, University of Melbourne, Parkville, Victoria 3010 Australia; 2 Laboratory of Liver Infectious Diseases, Faculty of Medicine and Health Sciences, Ghent University, De Pintelaan 185, 9000 Ghent, Belgium; 3 Burnet Institute, Melbourne, Australia; 4 Department of Surgery, The University of Adelaide and The Basil Hetzel Institute for Translational Health Research, Adelaide, South Australia, Australia; 5 Department of Pathology, Stanford University School of Medicine, Stanford, CA USA; 6 Department of Microbiology, Monash University, Clayton, Australia

## Abstract

The significant public health problem of Hepatitis C virus (HCV) has been partially addressed with the advent of directly acting antiviral agents (DAAs). However, the development of an effective preventative vaccine would have a significant impact on HCV incidence and would represent a major advance towards controlling and possibly eradicating HCV globally. We previously reported a genotype 1a HCV viral-like particle (VLP) vaccine that produced neutralizing antibodies (NAb) and T cell responses to HCV. To advance this approach, we produced a quadrivalent genotype 1a/1b/2a/3a HCV VLP vaccine to produce broader immune responses. We show that this quadrivalent vaccine produces antibody and NAb responses together with strong T and B cell responses in vaccinated mice. Moreover, selective neutralizing human monoclonal antibodies (HuMAbs) targeting conserved antigenic domain B and D epitopes of the E2 protein bound strongly to the HCV VLPs, suggesting that these critical epitopes are expressed on the surface of the particles. Our findings demonstrate that a quadrivalent HCV VLP based vaccine induces broad humoral and cellular immune responses that will be necessary for protection against HCV. Such a vaccine could provide a substantial addition to highly active antiviral drugs in eliminating HCV.

## Introduction

The introduction of directly acting antiviral agents (DAAs) has had a major impact on curing hepatitis C. However, reinfection remains a problem occurring in up to 27% of individuals who relapse to injecting drug use (IDU) after treatment within 8 years of achieving a SVR^1^. In addition, clearance of HCV infection does not necessarily result in protection against reinfection^2–5^ and thus the expectation of controlling HCV infection with DAAs alone is not realistic^6^. Furthermore, the widespread introduction of DAAs has been predicted to only halve the prevalence of chronic hepatitis (CHCV) and its complications^6–8^. Even a 16-fold increase in treatment rates will be insufficient to eliminate HCV, especially in high prevalence populations^6–8^. An effective vaccine will provide a substantial step towards controlling and possibly eradicating HCV globally^9,10^. In addition, vaccination after successful treatment with DAAs would also be expected to have a significant effect on reducing reinfection and HCV prevalence^10^.

Several lines of evidence support the importance of neutralizing antibodies in preventing HCV. Broadly neutralizing antibodies (bNAb) to viral E2 glycoprotein are protective and associated with HCV clearance^11–17^. In humans, the early induction of bNAb contributes to control of viraemia, resolution of infection^18^ and protection against persistent infection with HCV^19^. Vaccination of chimpanzees with mammalian cell-derived recombinant HCV gpE1E2 has been shown to prevent the development of persistent infection after homologous or heterologous virus challenge and in humans results in both bNAb and CD4^+^ T cell responses^20^. Furthermore, immunization of human volunteers with recombinant gpE1E2 results in the development of NAb responses^21^. A HCV E2 glycoprotein and retroviral Gag pseudotypic particle vaccine has been shown to induce high-titre bNAb in mice and macaques^22^. Chimeric hepatitis B-HCV viral-like particles (VLPs) containing E1E2 heterodimers of genotype 1a HCV have also been shown to elicit bNAb responses against heterologous HCV genotypes^23^. Finally, serum isolated from mice immunized with inactivated infectious cell culture derived HCV (HCVcc) protected human liver chimeric mice against homologous HCV challenge^24^. Strong T cell responses are also important but may insufficient on their own to prevent or clear HCV infection^25^.

We previously reported a human liver cell-derived genotype 1a HCV VLP vaccine that resulted in the induction of NAb and T cell responses to HCV^26,27^. The HCV VLPs have been characterised biochemically and shown to have a morphology typical of HCV virions^27–29^. Similarly, a genotype 3a HCV VLP vaccine also induces broad humoral and cellular immune responses^30^. In an approach similar to the human papilloma virus vaccine^31^ we have produced a quadrivalent genotype 1a/1b/2a/3a HCV VLP vaccine and established methods for the large-scale production and purification of this vaccine to ensure that immune responses to the vaccine will be HCV specific^32^. In this study, we show that our quadrivalent vaccine with and without adjuvant produced strong antibody, NAb, memory B and T cell responses in vaccinated mice. The addition of adjuvants improved granzyme B responses compared to vaccine alone. Moreover, select neutralizing human monoclonal antibodies (HuMAbs) targeting conserved antigenic domain B and D epitopes of the E2 protein^33–37^ bound to the HCV VLPs strongly, indicating that these critical epitopes are expressed on the surface of the particles. Overall, our results show that a quadrivalent HCV VLP vaccine produces broad HCV specific immune responses, which is encouraging for the development of a protective vaccine.

## Results

### Induction of HCV VLP specific antibody responses

In our previous work we demonstrated that genotype 1a HCV VLPs were able to produce strong humoral immune responses in mice^26,27^. We also recently reported the biochemical characterisation and methods for scaled-up production of the genotype 1a, 1b, 2a and 3a quadrivalent HCV VLP vaccine that was used in the current study^32^. To determine the immunogenicity of the quadrivalent vaccine we inoculated mice with a cocktail comprised of 20 µg of each genotype-specific HCV VLP in PBS or in combination with well-characterised adjuvants (alum, montanide or CFA) to establish the optimum adjuvant and to investigate the immunogenicity of our vaccine in the absence of adjuvant

**Figure 1:**
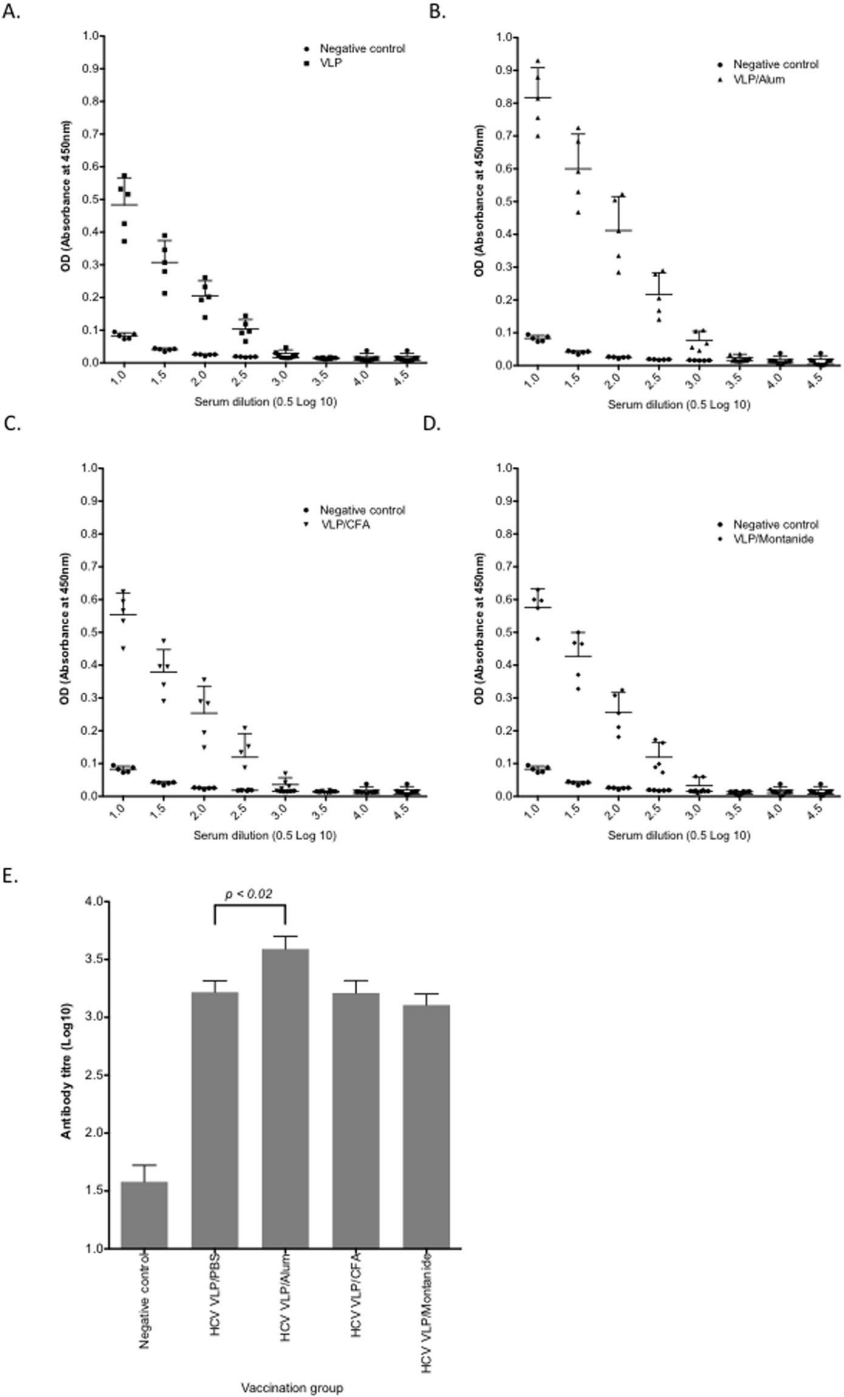
VLP-specific antibody responses elicited by immunization with quadrivalent vaccines. BALB/c mice (n = 5/group) were immunized subcutaneously at the base of the tail with 80 μg of quadrivalent VLP in PBS (**A**), or quadrivalent VLP combined with Alum (**B**), CFA (**C**) or Montanide (**D**). Negative control mice were immunized with vaccine diluent (PBS). Antibody levels in sera prepared from blood taken on day 21 were determined by ELISA. VLP-specific antibody titres for each immunization group were also determined (**E**). In all panels, individual animals are presented for each group, with the mean value being represented by the horizontal bar.

**Figure 2:**
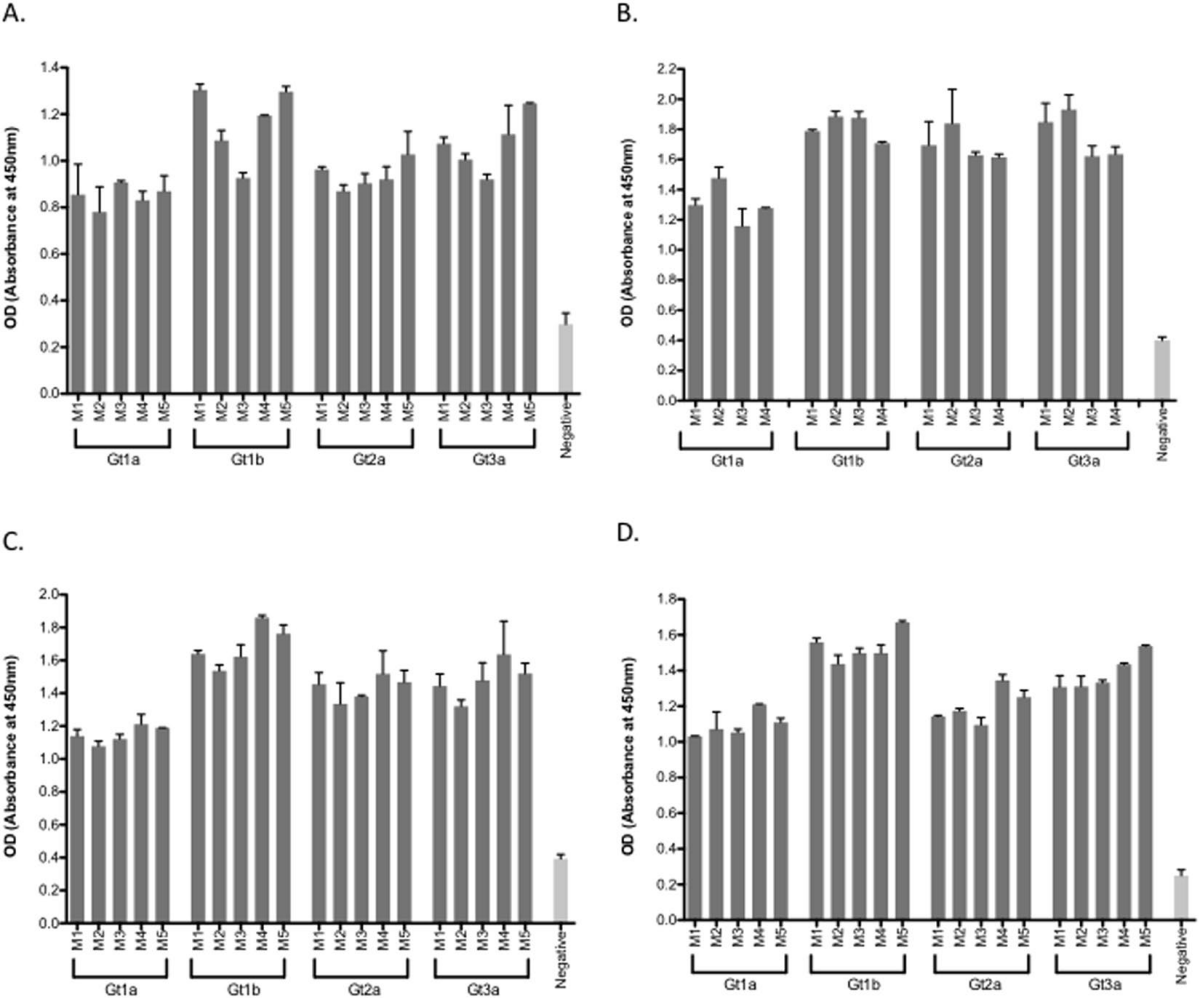
Genotype-specific antibody responses elicited by immunization with quadrivalent vaccines. BALB/c mice (n = 5/group) were immunized subcutaneously at the base of the tail with 80 μg of quadrivalent VLP alone (**A**), or quadrivalent VLP combined with Alum (**B**), CFA (**C**) or Montanide (**D**) and genotype specific antibody in sera prepared from blood taken on day 21 were determined by ELISA using genotype 1a, 1b, 2 or 3a HCV VLP as coating antigens. Negative control mice were immunized with vaccine diluent (PBS).

Mice were inoculated with two doses of quadrivalent vaccine two-weeks apart and sacrificed 1 week after the boost. Sera were prepared and tested by ELISA to determine antibody binding and anti-VLP antibody titre. All groups developed antibody responses, with the highest titre in the alum group (Fig. 1A–D). The antibody response to the vaccine in PBS was comparable to vaccine supplemented with either montanide or CFA (p = ns). The geometric mean titre for anti-HCV VLP antibody was highest in mice receiving the quadrivalent HCV VLPs in alum (3.58 log_10_ [95%CI 3.29 to 3.90]) compared to HCV VLPs in CFA (3.20 log_10_ [95%CI 2.92 to 3.52] p = 0.06), HCV VLPs in montanide (3.10 log_10_ [95%CI 2.85 to 3.38], p = 0.006) and HCV VLPs in PBS (3.21 log_10_ [95%CI 2.95 to 3.49] p < 0.023) (Fig. 1E). Mice in all vaccination groups also developed genotype specific antibody responses (Fig. 2). The strongest responses were consistently detected against HCV genotype 1b VLPs while responses to genotype 1a VLPs were lowest in all groups (Fig. 2). However, antibody responses to individual genotypes were of low titre.

### Neutralizing antibody responses to HCV VLP vaccine

**Figure 3:**
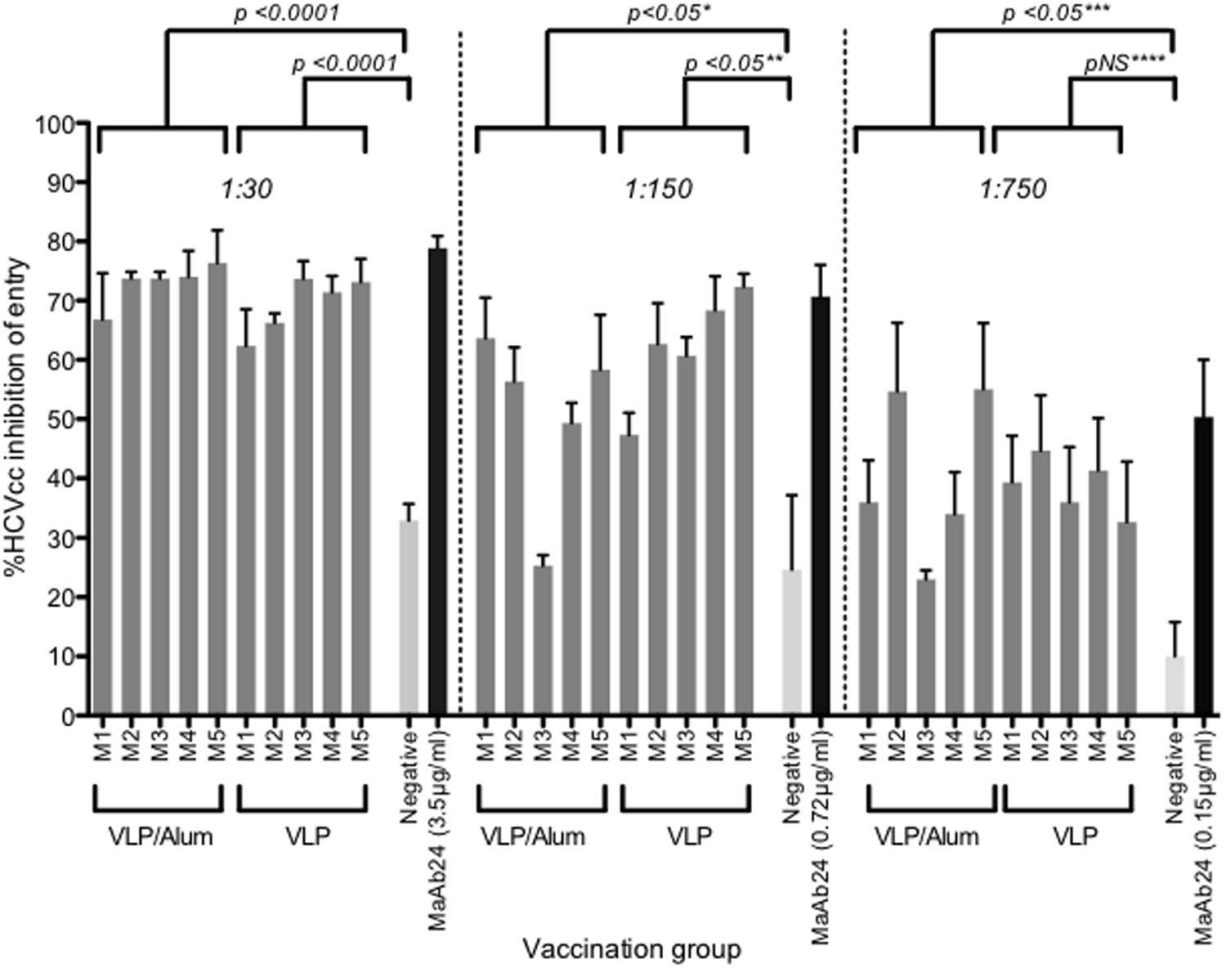
Neutralizing antibody responses to quadrivalent VLP alone or combined with alum. Percentage HCV inhibition was determined by preincubating HCV infectious cell culture virus (HCVcc) with an equal amount of diluted immune serum from mice inoculated with quadrivalent VLP combined with and without alum. Five-fold serial serum dilutions (1:30, 1:150 and 1:750) were tested. The concentrations (µg/ml) of the MAb24 used for each dilution series are indicated on the X-axis. Individual animals are presented for each group, with the mean value being represented by the horizontal bar. (*M3/VLP/1:150, p = ns; **M1/Alum /1:150, p = ns; ***M3/Alum/1:750, p = ns; ****M5/VLP/1: 750, p = ns).

Having shown that quadrivalent vaccine preparations could produce strong antibody responses we then determined NAb responses using cell culture derived Jc1 HCV^38^. Increasing dilutions of sera from mice immunized with the quadrivalent vaccine in PBS and with alum were compared for their ability to neutralize HCVcc. The sera from the mice immunized with HCV VLPs in alum inhibited HCVcc entry by 72.9% (+/−SD 3.6%) compared to 69.4% (+/−SD 4.9%) with VLPs in PBS and 32.9% (+/−SD 4.9%) with non-immune sera (p < 0.0001) (Fig. 3). These results compared favourably with the monoclonal antibody MAb24 which inhibited HCVcc entry by 78.5% (+/−SD 3.6) (Fig. 3). The inhibition of entry was lower with increasing serum dilutions, although this remained comparable to the level of neutralization with an analogous dilution of the MAb24 positive control (Fig. 3). Limited sera prevented studies on the cross reactivity of the neutralizing antibody response, however we are now determining this following vaccination in a large animal model (see Discussion).

### Epitopes important for neutralization of VLP

**Figure 4:**
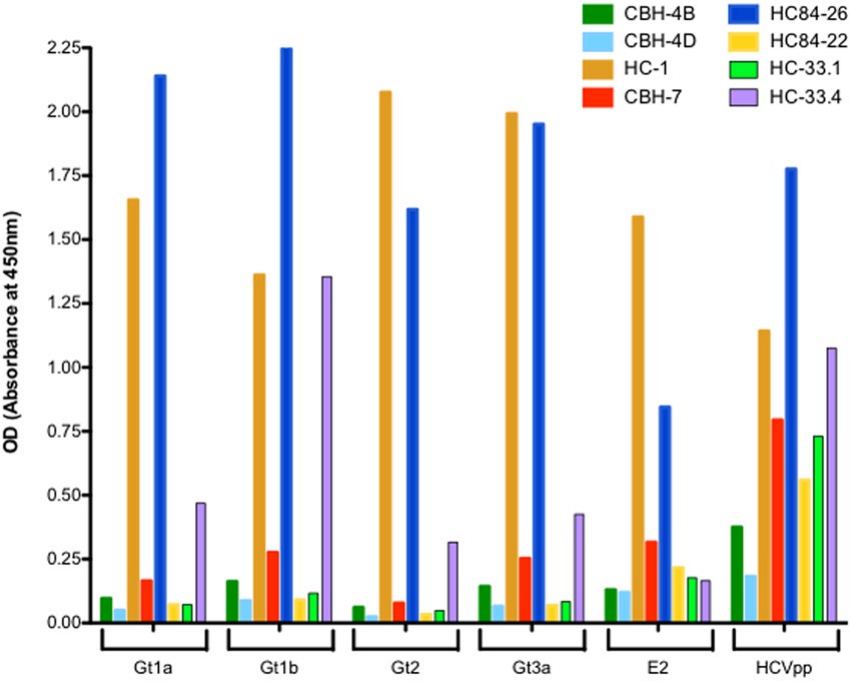
Binding of human monoclonal antibodies to VLP. (A) panel of human monoclonal antibodies (HuMabs) to conserved epitopes to antigenic domains A (CBH-4B and CBH-4D), (**B**) (HC-1), (**C**) (CBH-7), (**D**) (HC84.22 and HC84.26) and E (HC33.1 and HC33.4) on the E2 protein were tested by ELISA for their ability to bind to genotype specific VLP (Gt1a, 1b, 2 and 3a). The HuMabs were also tested for binding to soluble HCV E2 protein and genotype HCV pseudoparticles (HCVpp) by ELISA.

To gain further insight into epitopes that are important for the observed HCV neutralization by our quadrivalent vaccine, we used a panel of monoclonal antibodies directed at the E2 glycoprotein, the primary target of human NAb^33–35^. This panel consisted of HuMabs known to bind to the neutralizing face of E2 (HC-1, HC33.1, HC33.4, HC84.22 and HC84.26) and the non-neutralizing face (non-neutralizing CBH-4B and CBH-4D, and neutralizing CBH-7)^36^. Strong antibody binding to all genotype specific HCV was observed with NAb HC84-26 and HC-1 (Fig. 4). This binding pattern was also observed with binding to the E2 protein. Antibody HC-33.4 showed strong binding to genotype 1b and modest binding to genotypes 1a, 2a and 3a. With the exception of CBH-7, which showed low binding to genotypes 1a, 1b and 3a, all other antibodies tested failed to show specific binding (Fig. 4). Antibodies HC.1, CBH-7, HC84.26, HC84.22, HC-33.1 and HC33.4 bound HCVpp, although the binding of HC84-26 and HC-1 was generally lower than to HCV VLPs (Fig. 4). Titration of the highest binding antibodies HC-1 and HC84-26 demonstrated strong binding of HC-1 to all four genotype HCV VLPs (EC_50_ 7.9, 6.93, 6.55 and 6.16 µg/ml respectively) while HC84.26 showed stronger binding to genotypes 1a and 2a (EC_50_ 8.9 and 12 µg/ml respectively) compared to genotypes 1b and 3a (EC_50_ 7.25 and 8.63 µg/ml respectively (Table 1). The binding of HC84.26 to genotype 2a HCV VLPs was also significantly stronger compared to HC-1 (EC_50_ 12 µg/ml compared to 6.55 µg/ml) (Table 1).

**Table 1:** Binding of HC.1 and HC84.26 HuMabs to HCV VLPs.

EC50	Gt1a (µg/ml) Mean (+/−SD)	Gt1b (µg/ml) Mean (+/−SD)	Gt2a (µg/ml) Mean (+/−SD)	Gt3a (µg/ml) Mean (+/−SD)
HC.1	7.9 (1.19)	6.93 (1.6)**	6.55 (2.2)*	6.16 (1.8)^†^
HC84.26	8.9 (2.9)	7.25 (2.1)***	12 (2.1)	8.63 (2.36)

EC50 was determined for the binding of HC.1 and HC84.26 to genotype specific HCV VLPs. Results represent the mean (+/−SD) of 5 separate ELISAs for each antibody and each HCV VLP genotype.

*HC.1 Gt2a compared with HC84.26 Gt2a, p < 0.05.

**HC-1 Gt1b HC.1 compared with HC84.26 Gt2a, p < 0.05.

^†^HC-1 Gt3a compared with HC84.26 Gt2a, p < 0.05.

***HC84.26 Gt1b vs Gt2a, p < 0.05.

### Antigen Specific B-cell responses

**Figure 5:**
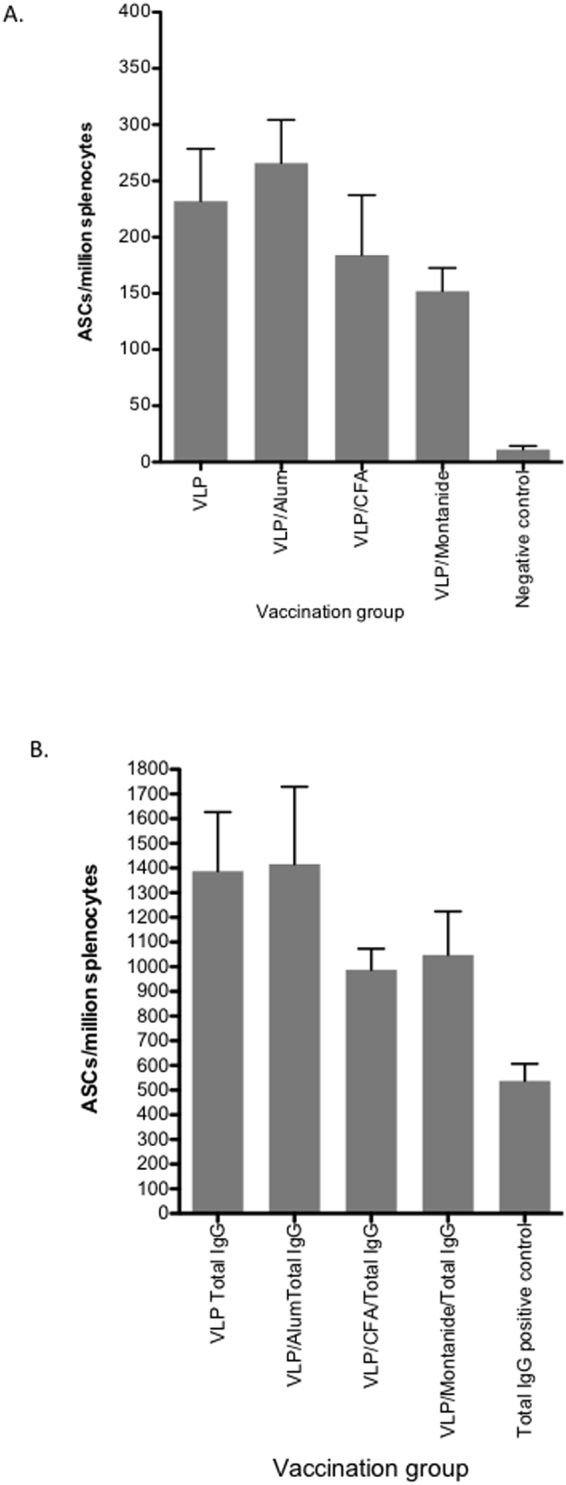
B cell responses and total IgG secretion following immunization with quadrivalent vaccines. BALB/c mice (n = 5/group) were immunized subcutaneously at the base of the tail with PBS alone (control) or 80 μg of quadrivalent VLP. Splenocytes were harvested on day 21 and frequencies of total HCV specific antibody secreting cells (ASCs) in each group (PBS, or vaccine combined with Alum, CFA or Montanide) (**A**) and total IgG (**B**) were determined by B cell ELISpot assay. The y-axis shows the antibody secreting cell (ASC) number/million splenocytes. Individual animals are presented for each group, with the mean value being represented by the horizontal bar.

**Figure 6:**
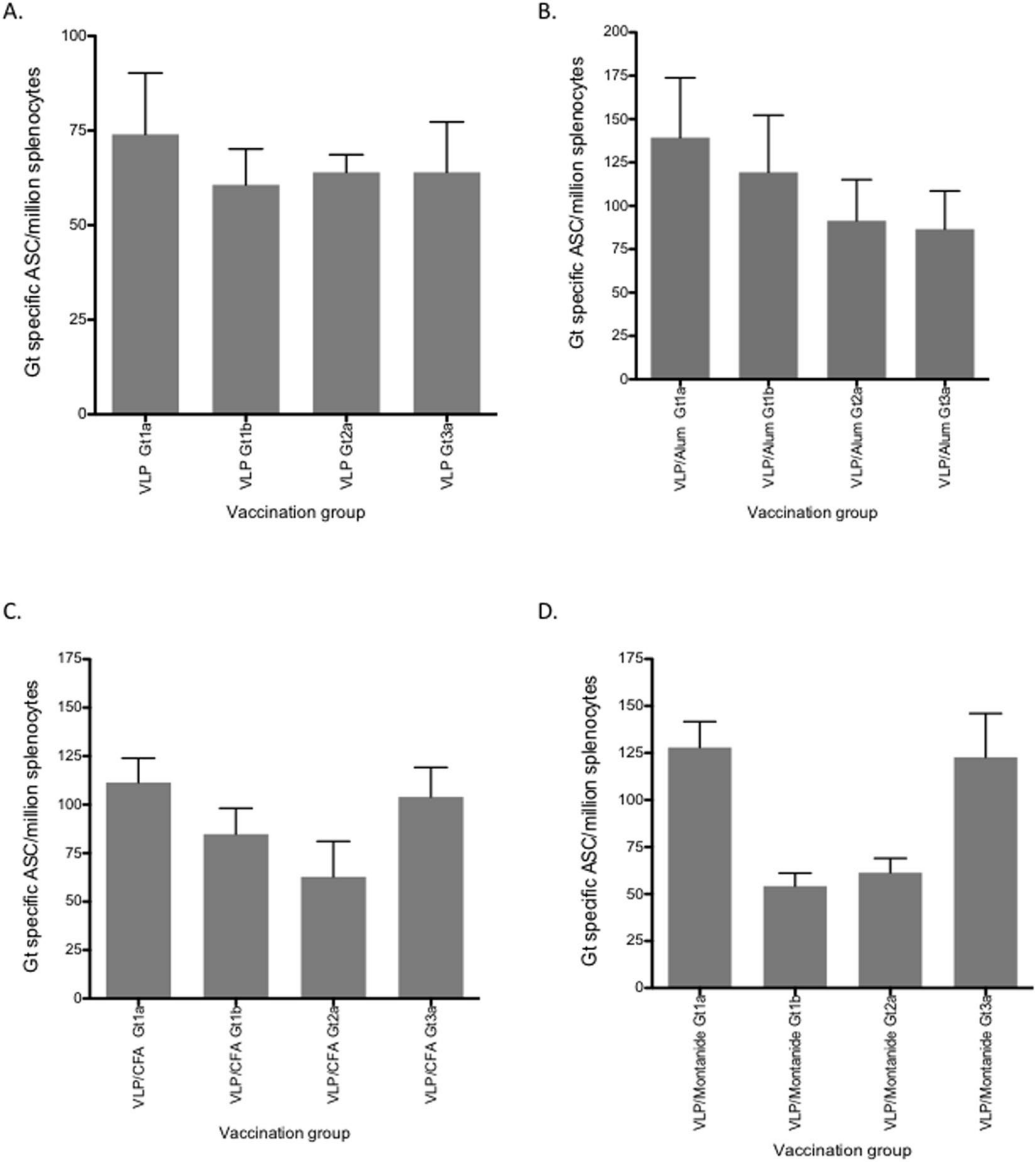
Genotype specific B cell responses following immunization with quadrivalent vaccines. BALB/c mice (n = 5/group) were immunized subcutaneously at the base of the tail with PBS alone (control) or 80 μg of quadrivalent VLP. Splenocytes were harvested on day 21 and frequencies of HCV genotype specific ASCs were determined for each group of mice immunized with quadrivalent HCV VLP vaccine in PBS (**A**) or combined with Alum (**B**), CFA (**C**) or Montanide (**D**). The y-axis shows the antibody secreting cell (ASC) number/million splenocytes. Individual animals are presented for each group, with the mean value being represented by the horizontal bar.

Induction of B cell responses following vaccination is important for long-term immunity against viral infection. We therefore assessed B-cell responses following vaccination with quadrivalent VLP by ELISpot. Initially we determined the number of antibody secreting cells (ASC) from splenocytes of mice vaccinated with quadrivalent VLP without adjuvant. This resulted in a mean number of 232 (+/−SD 104) ASC/million splenocytes (Fig. 5A). VLP with alum only resulted in a modest increase in ASC numbers (mean 266 (+/−SD 85.62)/million splenocytes) compared to VLP alone. Lower numbers of ASCs were observed following vaccination of VLPs adjuvanted with CFA or montanide (mean 184 (+/−SD 119.3) /million and 152 (+/−SD 46)/million splenocytes, respectively) (Fig. 5A). The total number of IgG ASCs for each of the vaccination groups following CpG stimulation is shown in Fig. 5B. We then determined the number of ASCs for each specific genotype and observed a similar response across all genotypes following vaccination with HCV VLPs alone (mean range 64 to 74 ASC/million splenocytes) (Fig. 6A). VLPs adjuvanted with alum showed an increase in numbers for each of the specific HCV genotypes (mean range 86–139/million) (p = ns) (Fig. 6B). Genotype specific responses were similar in both the CFA and montanide groups (mean range 62–111/million and 54–128/million (Fig. 6C,D respectively), although the lowest responses were observed for genotypes1a and 2a in the montanide group.

### Measurement of antigen-specific T-cell and Granzyme B responses elicited by quadrivalent vaccine

**Figure 7:**
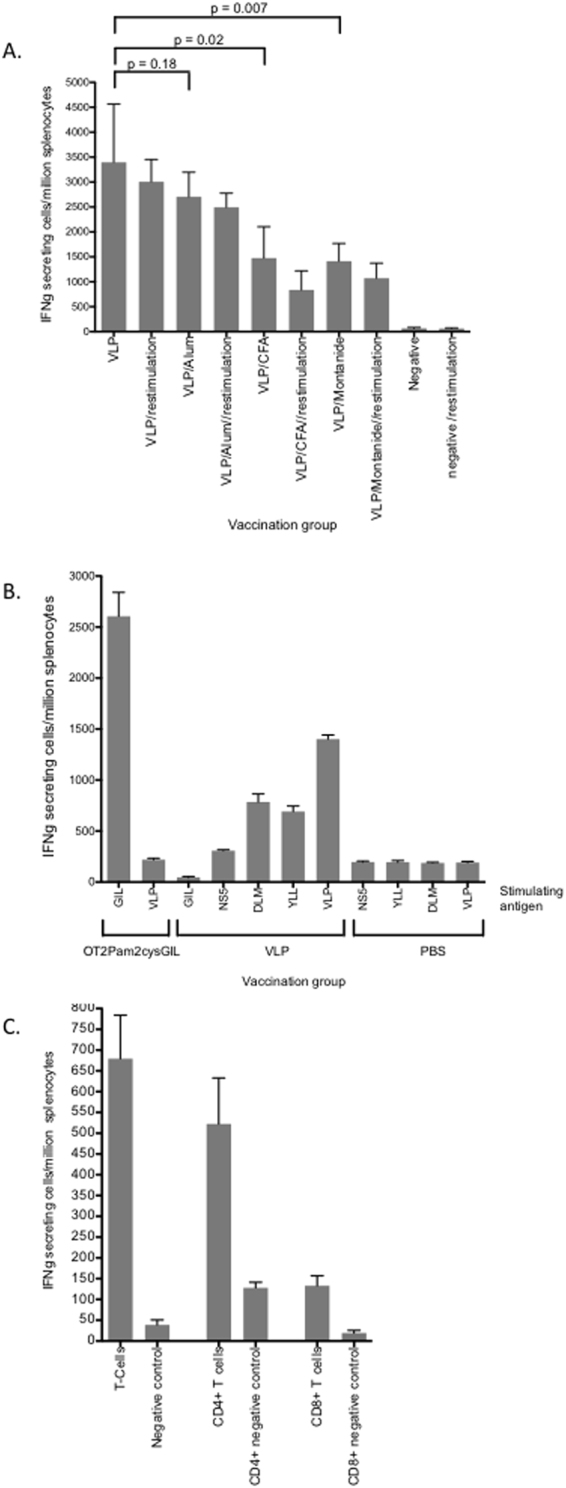
IFNγ T cell responses following immunization with quadrivalent vaccines. (**A**) BALB/c mice (n = 5/group) were immunized subcutaneously at the base of the tail with the quadrivalent VLP combinations as shown. Frequencies of IFNγ secreting cells before and after restimulation were determined by ELISpot assay. (**B**) HHD mice were vaccinated with OT2 Pam2Cys GIL, HCV VLPs or PBS and frequencies of IFNγ secreting cells were determined by ELISpot after stimulation with GILGFVFTL, HCV VLP, HCVcore_132_−140_ DLMGYIPLV (DLM), HCVcore_35–44_ YLLPRRGPRL (YLL) or the irrelevant HCV NS5B_2594–2602_ peptide. (**C**) T cells from BALB/c mice vaccinated with quadrivalent VLP alone were further purified from spleen using Miltenyi Biotec columns and CD4 (L3T4) and CD8a (Ly-2) microbeads and analysed by ELISpot assay. The mean value and standard deviation is shown for each treatment group.

As interferon-gamma (IFNγ) is crucial for immunity against intracellular pathogens we wished to determine if our quadrivalent vaccine could induce IFNγ responses following vaccination. The mean number of T cells secreting IFNγ following vaccination of mice with VLP alone was 3396 (+/−SD 1168) cells/million splenocytes. This was similar to the number observed following vaccination with VLP and alum (mean 2706 (+/−SD 1098) /million splenocytes, p = ns) (Fig. 7A). In contrast, T cell responses following vaccination of VLPs adjuvanted with either CFA or montanide were significantly lower than those after vaccination with VLPs alone (mean number 1476 (+/−SD 1404)/million splenocytes (p = 0.02) and 1416 (+/−SD 788)/million splenocytes (p = 0.007) respectively) (Fig. 7A). Restimulation for 5 days with 20 µg of quadrivalent VLP (5 µg/genotype) did not increase the number of IFNγ secreting T cells for any of the quadrivalent vaccination groups (Fig. 7A), suggesting that vaccination with VLPs alone resulted in maximal induction of VLP specific T cell responses.

To investigate the specificity of the CD8+ T cell response we vaccinated HHD mice with HCV VLPs, PBS or the lipopeptide, OT2Pam2CysGIL followed by stimulation of isolated splenocytes with GILGFVFTL, HCV VLP, HCVcore_132_−140_ DLMGYIPLV (DLM), HCVcore_35–44_ YLLPRRGPRL (YLL) or the irrelevant HCV NS5B_2594–2602_ peptide and analysed IFNγ responses by ELISpot. The mean number of CD8+ T cells secreting IFNγ following vaccination of mice with OT2 Pam2CysGIL and stimulation with GILGFVFTL was 2607 (+/−SD 116.8) cells/million splenocytes compared to 220 (+/−SD 24) cells/million splenocytes following HCV VLP stimulation (p < 0.0001)(Fig. 7B). In contrast, in the HCV VLP vaccinated group, the mean number of CD8+ T cells secreting IFNγ following stimulation with HCV VLP was 1401 (+/−SD 71), 786 (+/−SD 110) with DLM peptide, 692 (+/−SD 94) with YLL peptide compared to 300 (+/−SD 14) with NS5 (p = 0.001 vs HCV VLP) and 46 (+/−SD16) with GIL (p = 0.0001 vs HCV VLP) peptides (Fig. 7B). The IFNγ responses in PBS vaccinated control mice were poor against all stimulating antigens (Fig. 7B).

We then examined IFNγ secretion from purified CD4^+^ and CD8^+^ T cells derived from splenocytes from mice vaccinated with VLP alone. IFNγ secretion was observed from both cell types, however the number of IFNγ secreting CD4^+^ T cells was significantly higher than the number of IFNγ secreting CD8^+^ T cells (mean number 522 (+/−SD 221)/million splenocytes and 133 (+/−SD 47)/million splenocytes respectively) (Fig. 7C).

**Figure 8:**
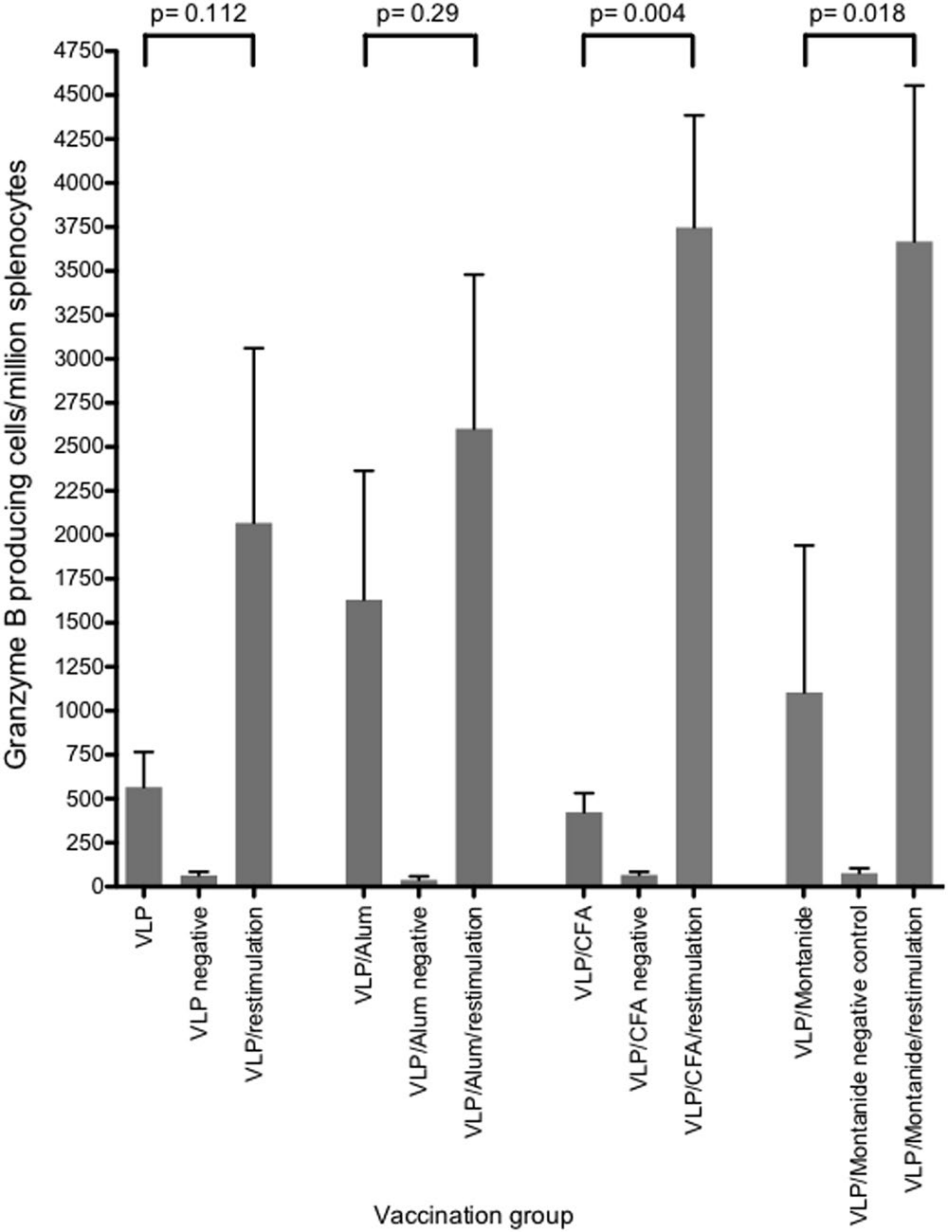
Granzyme B responses following immunization with quadrivalent vaccines. BALB/c mice (n = 5/group) were immunized subcutaneously at the base of the tail with the quadrivalent VLP combinations as shown. Splenocytes were harvested on day 21 and frequencies of Granzyme (**B)** responses before and after restimulation were determined by ELISpot assay. The mean value and standard deviation is shown for each treatment group.

To determine antigen specific cytotoxic T cell responses following vaccination with the quadrivalent HCV VLP vaccine we used a granzyme B assay which provides a reliable and sensitive in vitro measurement of cytotoxic CD8^+^ T cell responses^39^. The mean number of granzyme B producing cells following vaccination with VLP alone was 566 (+/−SE 200)/million splenocytes, which was similar to that observed from the VLP plus CFA group (mean number 424 (+/−SE 108)/million splenocytes) but significantly lower than the mean number observed following quadrivalent vaccination with VLP plus alum (mean number 1632 (+/−SE 731)/million splenocytes) or VLP plus montanide (mean number 1105 (+/−SE 836)/ million splenocytes (Fig. 8). Restimulation with quadrivalent VLP for 5 days resulted in an increase in the mean number of granzyme B producing cells in all vaccination groups, particularly in the VLP/CFA (mean number 3748 (+/−SE 637)/million splenocytes) and montanide (mean number 3668 (+/−SE 885)/million splenocytes) groups, but also in the VLP alone (mean number 2069 (+/−SE 992)/10^6^ splenocytes) and in the VLP plus alum group of mice (mean number 2604 (+/−SE 876)/10^6^ splenocytes) (Fig. 8).

## Discussion

In countries where modern antiviral therapies are now widely available there are estimated to be a large number of individuals who are unaware that they are infected, leaving a large residual pool of chronically infected individuals acting as a persistent source for ongoing HCV transmission^6^. An effective preventative vaccine would therefore have a significant impact on HCV prevalence.

We have shown that a mammalian cell-derived quadrivalent VLP vaccine results in both humoral and cell-mediated immune responses. The vaccine was found to be strongly immunogenic after just two doses, even in the absence of adjuvant. Although the quadrivalent VLP vaccine combined with alum produced higher overall antibody titres and memory B cell responses compared to VLPs alone or adjuvanted with montanide or CFA this did not translate to significantly stronger NAb responses. The quadrivalent vaccine also produced strong genotype specific memory B cell and antibody responses.

A vaccine derived from a single genotype can elicit NAb, however as shown with human papilloma virus vaccine the inclusion of antigens of a number of different genotypes could be expected to produce broader cross NAb responses^31^. This was highlighted by a recent study examining cross-genotypic immunity in a cohort of people who cleared but were subsequently reinfected with HCV. This report demonstrated that immunity against one HCV genotype did not confer immunity against heterologous genotypes, arguing that a vaccine including multiple genotypes could offer broader protection against HCV^40^. Broad HCV NAb are mainly directed to the E2 protein but there is also evidence showing that bNAbs may act by recognising native E1E2 heterodimers and not E2 alone^41,42^. An effective vaccine should therefore express native E1E2 structures. The importance of the neutralizing regions on the viral envelope is further highlighted by the ability of passive immunization with polyclonal and monoclonal antibodies directed to these regions to protect human liver chimeric uPA/SCID mice against challenge with human serum derived HCV^15^. The development of cross-NAb to epitopes on the surface of HCV that develop in the course of natural infection provides further encouragement for the development of a neutralizing HCV vaccine^43^. These findings are significant for HCV vaccine design because they reinforce the need to present both E1 and E2 proteins in the correct structural organisation as presented on virions or HCV VLPs. Furthermore, as HCV-specific NAbs recognize tertiary or quaternary structures^42^, the particulate structure of HCV VLPs and their ability to present conformational epitopes in their native state makes HCV VLPs an attractive vaccine candidate^22,23,26^.

The E2 protein contains the major conformational neutralizing antigenic regions organized in distinct clusters of overlapping epitopes (antigenic domains A-E or the AR3 and AR4 regions)^33,34,42^. Antibodies against these epitopes are broadly cross-neutralizing and protect human liver chimeric uPA/SCID mice against challenge with human serum derived HCV^42^. At least four distinct clusters of overlapping epitopes mediating broad virus neutralization have been described^33–35^. Recent crystallographic studies of the E2 core domain in complex with key NAbs have provided important insights in to the HCV envelope structure^44,45^. It has been possible to define both a neutralizing and a non-neutralizing face to the E2 core antigenic surface^44^. Together with comprehensive alanine substitution studies it has also been possible to map the binding of HuMabs to these critical regions of E2 with the most potent antibodies binding to domains B and D of the protein^36^. HuMabs that are known to be potently neutralizing for HCV bound strongly our HCV VLPs. Two of these antibodies, HC84.26 and HC-1, bind to epitopes in domains B and D on the neutralizing face of the E2 protein, indicating that these important epitopes are present and accessible on the surface of our HCV VLPs. HC84.26 bound more strongly to genotype 2a followed by 1a and 3a HCV VLPs. In contrast, HC.1 bound strongly to all four genotypes HCV VLPs, suggesting that there may be differences in the epitope sequences or presentation on the different genotype VLPs. One of the other NAbs that we tested, HC33.4 showed strong binding to genotype 1b, but modest binding to the other HCV genotypes, whereas the other neutralizing antibody HC33.1, which is directed to a mostly linear epitope, did not bind to any of the HCV genotypes. The reason(s) for this are unclear. However, the overriding message from our data is that crucial epitopes important for neutralization of HCV are expressed on our VLPs. This information provides critical data for the design of HCV NAb vaccines. The complex nature of how neutralizing epitopes are presented on the surface of HCV suggests that these important antigenic regions need to be closely reproduced in a vaccine.

HCV VLPs also offer an approach to elicit cellular immune responses in addition to NAb in a single vaccine platform^23,26,30,27^. Compared to HCV core, E1 and E2 DNA vaccines HCV VLPs produce stronger cytotoxic T cell responses in mice^46^. This is important given the role of HCV core specific T cell responses in clearance of HCV^47^. Our quadrivalent vaccine produced strong CD4^+^ T cell and granzyme B responses. These are important findings as the spontaneous clearance of HCV, control of HCV viremia and the development of HCV specific memory B cell responses are all associated with the development of early and multi-specific class I CD8^+^ and class II CD4^+^ T cell responses in addition to NAb responses^48–50^. Our results are encouraging as they show that a quadrivalent HCV VLP vaccine can induce broad immune responses that will be required for protection against HCV.

Our data demonstrate that a quadrivalent HCV VLP based vaccine, in the absence of adjuvant, is able to produce broad humoral and cellular immune responses that are necessary for protection against HCV infection. An HCV VLP based vaccine would fulfil the requirement of delivering critical conformational neutralizing epitopes in addition to providing HCV core specific CD4^+^ and CD8^+^ epitopes and could provide a substantial addition to highly active antiviral drugs in the overall goal to eliminate HCV.

## Methods

### Production and purification of HCV VLPs and adjuvants

Construction of recombinant adeno-encoding HCV structural proteins core E1 and E2 and the large scale production, purification and characterisation of the quadrivalent genotype 1a, 1b, 2a, and 3a HCV VLP vaccine used in this study has been reported previously^26,27,32^. Human monoclonal antibodies (HuMabs) to conserved epitopes on E2 to antigenic domains A (CBH-4B and CBH-4D), B (HC-1), C (CBH-7), D (HC84.22 and HC84.26) and E (HC33.1 and HC33.4) are as described^33–37^. MAb24 is a murine NAb that recognizes a linear epitope in the E2_661_ protein that lacks the HVR1, HVR2 and igVR regions^51^.

### Mice and immunization protocol

All animal experiments used 8- to 12-week old BALB/c mice housed in the Doherty Resource Facility under specific pathogen-free conditions. Animal experiments were approved and performed in accordance with the conditions as specified by The University of Melbourne’s animal ethics committee. Six Groups of five age and weight matched BALB/c mice were immunized subcutaneously on each side of the base of tail (50 µl per dose) with PBS alone (negative control) or 80 μg of quadrivalent VLP with or without adjuvant (Alum, CFA or Montanide). Two weeks later the mice received a second equivalent dose of quadrivalent vaccine. Animals were sacrificed one week after the final immunization, spleens were harvested and blood was collected for serum preparation. To determine the specificity of the T cell response to the HCV VLPs HLA-A2kb transgenic mice (HHD) were vaccinated with HCV VLPs or PBS subcutaneously as above or intranasally with 50 µl (25nmol) of the lipopeptide (OT2 Pam2CysGIL) on days 0 and 9. This vaccine contains the epitope GILGFVFTL (M1_58–66_) derived from the matrix 1 protein of influenza A virus linked to the ovalbumin-derived CD4+ T helper cell epitope OT2 (ISQAVHAAHAEINEAG) and induces CD8+ T cell responses in mice to serve as a positive control^52^. Animals were sacrificed one week after the final immunization and spleens were harvested.

### Enzyme-linked immunosorbent assay

Anti-HCV VLP antibody titres of antibody were determined by ELISA as previously described^26^. In brief, for VLP-specific antibody responses, flat bottom 96-well polyvinyl plates were coated with purified quadrivalent VLPs (20 µg/ml) in carbonate coating buffer (100 mM Na_2_CO_3_, and NaHCO_3_, pH 9.6) overnight at 4 °C. The plates were then blocked with 100 µl of BSA (10 mg/ml) in PBS and incubated for 2 hours at 37 °C before washing four times with PBST (PBS containing v/v 0.05% Tween-20. Serum, starting at a dilution of 1:10 was serially diluted 0.5-log_10_ in BSA (5 mg/ml) in PBST and incubated for 1 hour at room temperature. Identical conditions were also used for determination of genotype-specific antibody responses. For binding of HuMabs to genotype specific VLP, coating and blocking of the plates was as described above, with the exception of the latter being at room temperature. The HuMabs were diluted in PBS with BSA (5 mg/ml) at 5 µg/ml for 2 hours at room temperature. No Tween was added to wash buffers or diluents. Absorbance values were determined on a Labsystems Multiskan Multisoft plate reader at 450 nm. Titres of antibody are expressed as the reciprocal of the highest dilution of serum required to achieve an optical density of 0.2. The production of recombinant E2 and HCVpp for the HuMab binding assays have been described previously^26,38^.

### HCV Neutralization Assay

Neutralization assays against HCV infectious cell culture system (HCVcc) were performed by mixing HCVcc virus with an equal volume of serially diluted immune serum^53^. Each experiment was performed in triplicate. The virus/serum mixture was incubated for 1 h at 37 °C before addition to Huh7.5 cells that were seeded 24 h earlier at 30,000 cells/well in 48 well plates for 4 h. Cells were washed at least 4 times and replenished with fresh DMF10NEAA and incubated for a further 48–72 h. Luciferase activity was measured in clarified lysates using Renilla luciferase substrate (Promega) and a FLUOstar Optima microplate reader fitted with luminescence optics (BMG Life Technologies, Germany). The neutralization titre was calculated from 6-point dilution curves as the reciprocal dilution of serum to reduce luciferase activity by 50% (ID50). The data shown is the mean from at least two independent experiments. MAb24, used as a positive control for inhibition of HCVcc entry, is a murine NAb that recognizes a linear epitope in the modified recombinant E2_661_ protein that lacks the HVR1, HVR2 and igVR regions. The MAb24 antibody is produced using a Miniperm bioreactor, with a final concentration of the antibody of 150 µg/ml. The antibody is diluted 1 in 8 (18 µg/ml) and then used in a series of dilutions^51^.

### ELISpot Assays

#### Measurement of B Cell Specific Antibody Production

For the detection of specific antibody secreting cells by ELISpot, polyvinylidene fluoride (PVDF) membrane-lined 96-well plates (Mabtech, Nacka Strand, Sweden) were coated overnight at 4 °C with 100 µl of PBS containing quadrivalent VLPs (4 µg/well or 40 µg/ml), genotype specific VLPs of 1a, 1b, 2a and 3a, each at 1 µg/well or 10 µg/ml of anti-IgG antibody (10 µg/ml) as previously described^26,54^ except that biotinylated anti-IgG antibody and streptavidin-conjugated horse-radish peroxidase (both from Mabtech, Nacka Strand, Sweden) were used as detecting reagents. Memory B cells in splenocytes were stimulated to differentiate in to antibody secreting cells with the TLR2 agonist R848 and IL2 according to the manufacturers instruction (Mabtech, Nacka Strand, Sweden)^55^. Individual spots were counted using an AID EliSpot Reader (Strassberg Germany).

#### Detection of IFN-γ-secreting Cells

ELISpot assays were carried out using Mouse IFN-γ ELISpot ^BASIC^ (HRP) kit (Mabtech, USA) with PVDF membrane-lined 96-well plates (Millipore, Ireland) wells precoated with anti-IFN-γ capture monoclonal antibody AN18 at 1 mg/ml. Plates were then blocked with RF 10 medium. Splenocytes stimulated for 5 days with 20 µg total VLPs per well were harvested, washed and serial dilutions commencing at 1 × 10^5^ cells/ml added to the wells. Forty-eight hours later, plates were washed with PBS and biotinylated anti-IFN-γ capture antibody (clone R4-6A2-Mabtech USA) was added and incubated for 2 hours at room temperature in a humidified atmosphere. Plates were then washed and streptavidin conjugated horse-radish peroxidase (Mabtech, USA) was added and incubated for one hour. Spots representative of IFN-γ-producing cells were developed by the addition of TMB.

For HHD mice, splenocytes were stimulated with HCV VLPs (10 μg/ml), HCV core specific peptides; HCVcore_132_−140_ DLMGYIPLV (DLM) or HCVcore_35–44_ YLLPRRGPRL (YLL) (10 μg/ml) or an irrelevant HCV NS5B_2594–2602_ peptide (10 μg/ml) per well, harvested, washed and serial dilutions commencing at 1 × 10^5^ cells/ml added to the wells. Splenocytes from mice inoculated with OT2Pam2CysGIL were stimulated with the peptide GILGFVFTL (10 μg/ml). The ELISpot assay was performed as described above.

#### Detection of Granzyme B-secreting Cells

For the detection of Granzyme B-secreting cells, a Mouse Granzyme B ELISpot Development Module together with reagents from an ELISpot Blue Color Module (R&D Systems, Minneapolis, USA) including a Granzyme B positive control were used according to the manufacturer’s instructions. PVDF membrane-lined 96-well plates (Millipore, Ireland) were coated with anti-Granzyme B monoclonal antibody at 1 in 60 dilution. The following day, plates were washed with PBS and blocked (1%BSA, 5% sucrose in PBS) for 2 h at room temperature. Splenocytes stimulated for 5 days with VLPs were then added to the plate and incubated overnight at 37 °C. The plate was then washed and detection antibody added and incubated overnight at 4 °C. After washing the plates, Streptavidin-HRP was added to the wells at 1:1000 and incubated for one hour at room temperature. Spots were developed by the addition of TMB.

### Statistical analysis

Statistical analysis was performed using the Prism 5.0 software (GraphPad). In all cases the mean ± standard deviation of the mean (SD) is shown unless otherwise stated. P values for statistical analysis were calculated using one-way ANOVA. Differences were considered statistically significant when p values were less than 0.05 (p < 0.05) with a 95% confidence level.
